# Paddy ponding water quality responses to land use intensity in flooded rice systems in Uruguay

**DOI:** 10.1002/jeq2.70156

**Published:** 2026-02-27

**Authors:** G. Cantou, P. González‐Barrios, I. Furtado, A. Roel

**Affiliations:** ^1^ Departamento de Sistemas Agrarios y Paisajes Culturales, Centro Universitario Regional del Este Universidad de la República Treinta y Tres Uruguay; ^2^ Departamento de Biometría, Estadística y Computación, Facultad de Agronomía Universidad de la República Montevideo Uruguay; ^3^ Instituto Nacional de Investigación Agropecuaria (INIA) Treinta y Tres Uruguay

## Abstract

Understanding water quality responses in flooded rice (*Oryza sativa L*.) systems is essential to reconcile high productivity with environmental protection. This study provides the first high‐frequency dataset of paddy water quality dynamics in temperate South American rice systems, conducted in the Laguna Merín Basin (Uruguay–Brazil). Through detailed field‐scale monitoring under real management and objective fertilization criteria, we evaluated how land use intensity influences physicochemical variables and nutrient speciation in paddy ponding water under two contrasting systems: rice–pasture rotation (RP) and continuous rice (CR). Over two seasons, a biphasic temporal pattern was observed, with elevated phosphorus (P) and nitrogen (N) concentrations during early flooding (weeks 1–7) followed by stabilization. Despite both systems following best management practices, the more intensive CR consistently showed higher total and dissolved reactive P, while N concentrations were similar between systems and largely dominated by dissolved organic forms, indicating that N dynamics were governed by internal cycling rather than fertilizer inputs. Overall, land use intensity affected water quality in a nutrient‐specific manner: P was more responsive to management and enhanced mobilization under flooded conditions, whereas N appeared buffered by biogeochemical processes. The lower P enrichment observed in the RP rotation suggests that management practices associated with this system may help moderate nutrient concentrations in paddy water. This study provides field‐based evidence that differences in land use intensity influence nutrient behavior in flooded rice systems, contributing insights to improve water quality protection and sustainability in temperate rice agroecosystems.

## Core Ideas

- High‐frequency monitoring revealed phase‐specific water quality dynamics in flooded rice.
- Land use intensification increased P but not N concentrations in paddy water.
- The early flooding phase was the critical window for nutrient mobilization and mitigation efforts.
- Dissolved reactive P and organic N dominated nutrient fractions across systems.
- Including pasture periods in rotation mitigated P enrichment and eutrophication risk.

## Plain Language Summary

Rice fields are often flooded, which can move nutrients like phosphorus (P) and nitrogen (N) from soil into water, potentially affecting nearby rivers and wetlands. This study examined how different land use intensities influence water quality in rice fields in Uruguay, comparing a traditional rice–pasture rotation with a more intensive continuous rice system used elsewhere as a contrasting case. Over two growing seasons, water in rice paddies was monitored weekly for key nutrients, their chemical forms (speciation), and basic water quality indicators. We found that nutrient concentrations followed a two‐phase pattern: high N and P during the first 7 weeks after flooding, then stabilization. P was higher in the intensive system, while N remained similar across systems, mostly in dissolved organic forms, showing that internal cycling controlled N rather than fertilizer. Maintaining pastures can help protect water quality while sustaining rice production.

**Abbreviations**
CR: continuous rice
DIN: dissolved inorganic Nitrogen
DON: dissolved organic nitrogen
DOP: dissolved organic phosphorus
DRP: dissolved reactive phosphorus
DTN: dissolved total nitrogen
DTP: dissolved total phosphorus
EC: electrical conductivity
IW: irrigation water
LME: linear mixed‐effects
PCA: principal component analysis
PN: particulate nitrogen
PP: particulate phosphorus
PPW: paddy ponding water
RP: rice–pasture rotation
SE: standard error
SI: sustainable intensification
TN: total nitrogen
TP: total phosphorus
TSS: total suspended solids.

## 1 INTRODUCTION

Agricultural intensification remains a cornerstone of global food and fiber production, yet its environmental consequences continue to challenge nutrient management and water quality worldwide (Flaten et al., 2025; Paudel & Crago, 2021). Flooded rice systems represent a critical interface between agricultural productivity and aquatic ecosystems, where management practices directly influence nutrient cycling and losses (Cui et al., 2020; Krupa et al., 2011). Achieving sustainable intensification (SI) in these systems is particularly complex because their hydrological and biogeochemical conditions modify nutrient availability and mobility (Y. Feng et al., 2004).

Rice paddies (*Oryza sativa L*.), typically located in low‐lying landscapes with extensive irrigation networks, maintain a shallow water layer known as paddy ponding water (PPW). Losses of nitrogen (N) and phosphorus (P) from rice cultivation significantly contribute to freshwater eutrophication, with nutrient concentrations in PPW closely correlating with those in surface runoff (Cui et al., 2020; J. Liu et al., 2016). Because runoff events are episodic and difficult to capture, monitoring PPW over time provides an integrative way to assess nutrient loss risks and to guide mitigation strategies (Hua et al., 2017; S. Li et al., 2020). Understanding the chemical forms of N and P is therefore critical for designing targeted measures to control agricultural nonpoint‐source pollution (Yu et al., 2022). Incorporating the temporal variability of nutrient concentrations into management frameworks further allows identification of periods of greatest vulnerability, supporting adaptive practices that balance agricultural productivity with water quality protection.

Although numerous conservation practices, such as reduced tillage, cover crops, crop rotations, and nutrient management, have been promoted to mitigate nutrient export (Hussain et al., 2025; Sharpley et al., 2006), empirical evidence directly linking these practices to measurable water quality outcomes remains limited. Recent analyses emphasize the need for field‐based, process‐level studies that quantify how management intensity translates into nutrient losses across scales (Naslund et al., 2025). High‐frequency monitoring of PPW composition under contrasting land use intensities can bridge this gap and provide insights into short‐term nutrient mobilization in flooded systems.

Consistent with global trends (Yuan et al., 2021), Uruguay's rice production has expanded steadily, achieving internationally recognized yields that have increased by about 100 kg ha^−^^1^ annually over the past two decades (Tseng et al., 2020). Rice dominates the lower part of the Laguna Merín Basin, one of South America's largest freshwater systems and home to the UNESCO‐designated Bañados del Este Biosphere Reserve. Although native grasslands still dominate land cover, the rice sector accounts for 97% of recorded water use in the basin (Fabre et al., 2021). Unlike many global rice systems that rely on continuous rice (CR) or cereal rotations, Uruguay traditionally integrates rice with perennial pasture rotations. This system enhances soil quality and productivity while maintaining relatively low external inputs (Macedo et al., 2021).

However, recent intensification trends, shorter pasture periods, and inclusion of crops such as soybean (*Glycine max L*.), have raised environmental concerns (Macedo, Roel, Ayala, et al., 2022). To sustain yields, N topdressing rates have increased by about 3 kg ha^−^^1^ annually over the last two decades (Molina & Terra, 2023). While intensification has improved yield and resource‐use efficiency, it has also increased potential N losses by 37% (Pittelkow et al., 2016). CR systems with cover crops tend to show reduced N use efficiency and less stable environmental outcomes compared with rice–pasture (RP) rotations (Macedo, Roel, Velazco, et al., 2022). Together, these patterns emphasize that increasing fertilization rates and shortened rotations heighten the risk of N runoff and associated ecological impacts (X. Zhao et al., 2009).

P plays a pivotal role in freshwater degradation through eutrophication (Oliveira & Machado, 2013). In the Laguna Merín Basin, P concentrations often exceed regulatory limits, with agriculture accounting for nearly all nutrient inputs (Fabre et al., 2021). P levels are closely correlated with agricultural land extent, and long water residence times promote recurrent algal blooms (da Silva et al., 2019). Long‐term analyses of the lagoon's water and sediments reveal historical ecosystem shifts linked to agricultural intensification (Bueno et al., 2021). Despite this evidence, empirical data on nutrient dynamics in flooded rice fields under contrasting management intensities remain scarce, particularly in temperate South American systems.

Under flooded conditions, fertilizer‐derived nutrients interact with strongly reducing soil environments that fundamentally alter N and P cycling. Anaerobic conditions suppress nitrification, increasing the dominance of reduced N forms in PPW, while redox‐driven dissolution of Fe‐ and Al‐bound P can enhance P availability and mobility shortly after flooding (Cui et al., 2020; Y. Feng et al., 2004; Yan et al., 2023). In this context, nutrient dynamics in PPW are not only a function of fertilizer management, but also of application timing, hydrological management, and soil biological processes, which together determine both crop nutrient availability and losses via runoff.

Based on this context, our study examines nutrient concentrations and physicochemical dynamics in PPW under contrasting land use intensities in irrigated lowland rice systems. We compare the traditional RP rotation with a CR system, representing higher land use intensity. Both systems are part of a long‐term experimental platform aligned with SI principles. By analyzing temporal variability and nutrient speciation under field conditions, we aim to determine how production intensity influences nutrient cycling and potential water quality risks. We hypothesized that land use intensification would alter both the magnitude and timing of water quality variables in PPW, with CR showing higher and more variable concentrations of N and P than RP rotation. This integrative approach advances understanding of the interactions among land use intensity, water quality, and nutrient cycling in temperate rice systems, contributing insights relevant to SI strategies globally.

**Core Ideas**

- High‐frequency monitoring revealed phase‐specific water quality dynamics in flooded rice.
- Land use intensification increased P but not N concentrations in paddy water.
- The early flooding phase was the critical window for nutrient mobilization and mitigation efforts.
- Dissolved reactive P and organic N dominated nutrient fractions across systems.
- Including pasture periods in rotation mitigated P enrichment and eutrophication risk.

## 2 MATERIALS AND METHODS

### 2.1 Study area and experimental design

The study was conducted in a long‐term experiment at the INIA Paso de la Laguna Experimental Station (Treinta y Tres, Uruguay; 33°16′22.21″ S, 54°10′23.10″ W), within the Laguna Merín Basin. The humid mesothermic climate is characterized by a mean (± SD) daily temperature of 21.8 ± 3.0°C and 22.0 ± 3.3°C, and total rainfall of 667 and 530 mm during the 2020/2021 and 2021/2022 rice seasons, respectively (hereafter referred to as 2020 and 2021).

The experiment followed a randomized complete block design (three replicates, 1200 m^2^ plots) established in 2012 under continuous no‐tillage (Macedo et al., 2021). Two rice‐based rotations with contrasting land use intensities were evaluated. Here, land use intensification refers to increased cropping frequency and external input dependence required to sustain annual rice production. Accordingly, the two systems differed in their N and P fertilization requirements, which were inherent to each system rather than imposed as experimental treatments.

The selected systems were: (1) RP rotation (5‐year cycle): two consecutive rice crops interspersed with a winter cover of annual ryegrass *Lolium multiflorum* Lam., and a perennial pasture established after the second rice harvest and maintained for approximately 3.5 years. The pasture mixture included tall fescue *Festuca arundinacea* Schreb., white clover *Trifolium repens* L., and birdsfoot trefoil *Lotus corniculatus* L. and (2) CR (1‐year cycle): annual rice cultivation with a winter cover crop of Egyptian clover *Trifolium alexandrinum* L.

For the RP system, the first rice crop of the rotation was evaluated in both years. Two field replicates per treatment were monitored. Although the replication level was limited, this design enabled intensive, high‐frequency sampling across two consecutive seasons under comparable conditions, providing an initial assessment of water quality dynamics to guide future expanded monitoring. Soils are Typic Argialbolls with silty clay loam texture, with slopes <0.5% (Table 1).

**TABLE 1:** Mean and standard deviation for soil properties (0‐ to 15‐cm depth) at the beginning of each rice season (2020 and 2021) for continuous rice (CR) and rice–pasture rotation (RP) treatments.

		2020		2021	
Characteristic	Unit	CR	RP	CR	RP
Soil organic carbon	g kg^−1^	15.84 ± 0.46	17.72 ± 2.34	16.91 ± 0.28	16.78 ± 0.68
Total N	g kg^−1^	1.69 ± 0.03	1.84 ± 0.17	1.83 ± 0.06	1.77 ± 0.06
Total P	g kg^−1^	0.19 ± 0.003	0.21 ± 0.02	0.18 ± 0.03	0.21 ± 0.002
Available P content^a^	ug g^−1^	5.90 ± 0.58	13.71 ± 0.58	5.76 ± 0.55	9.39 ± 2.20
pH		5.95 ± 0.02	5.68 ± 0.02	5.85 ± 0.01	5.71 ± 0.11
Cation exchange capacity^b^	meq 100 g	12.61 ± 0.76	12.23 ± 0.76	12.97 ± 1.70	13.32 ± 0.05
Total exchangeable bases	meq 100 g	9.91 ± 0.76	9.17 ± 0.51	10.01 ± 1.56	10.27 ± 0.05
Base saturation	%	78.50 ± 1.30	75.00 ± 0.51	77.07 ± 1.97	77.08 ± 0.08

^a^ Determined by the citric acid extraction method.

^b^ At pH 7.

### 2.2 Crop management

**FIGURE 1:**
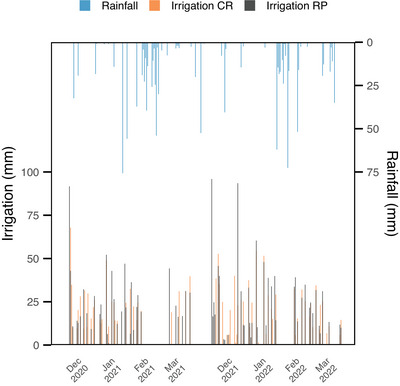
Daily rainfall and irrigation inputs during the rice flooding seasons (2020 and 2021) for continuous rice (CR) and rice–pasture rotation (RP) treatments.

The rice variety INIA Merín was direct‐seeded under no‐tillage in mid‐October (142 kg ha^−^^1^). Surface irrigation used water from the Olimar River, controlled with ultrasonic flow meters (DIEHL HYDRUS). Flooding began in late November and was maintained until 30 days after flowering (mid‐March). Irrigation inflow and rainfall were monitored (Figure 1) to document hydrological conditions required to maintain a constant floodwater depth (≈10 cm) across treatments, providing a consistent framework for comparing PPW quality. Total irrigation inputs averaged 719 ± 152 mm (RP) and 785 ± 21 mm (CR) in 2020, and 1049 mm ± 142 (RP) and 884 ± 2.9 mm (CR) in 2021.

Fertilization practices differed between treatments following standardized criteria defined at the onset of the long‐term INIA experiment, consistent with the contrasting land use intensity of each system (Aguirre‐Miguez et al., 2025). These criteria were designed to sustain long‐term productivity and anticipate potential soil nutrient constraints. In RP, P and N rates were adjusted following sufficiency‐level guidelines (Castillo et al., 2015; Hernández et al., 2013), whereas in CR, P fertilization followed a nutrient replacement approach and N rates were based on the estimated uptake for a 10 Mg ha^−1^ target yield (Castillo et al., 2015).

In both treatments, a basal compound fertilizer supplying P and K, along with a small amount of N (3 kg N ha^−^^1^ in 2020 and 7 kg N ha^−^^1^ in 2021), was applied in‐line at sowing. In the RP treatment, this basal application accounted for the total P (TP) input, whereas in CR, additional P was used by surface broadcasting at planting to reach the target total rate. Accordingly, TP application rates in RP were 15 and 20 kg P_2_O_5_ ha^−^^1^ in 2020 and 2021, respectively, whereas rates in CR were 85 and 92 kg P_2_O_5_ ha^−^^1^ in the same years (Table S1).

N was also applied by surface broadcasting as urea in two split applications: at tillering (V4–V6) on dry soil immediately before flooding and at panicle initiation (R0) on flooded soil. Total N inputs were 76 kg ha^−^^1^ in 2020 and 108 kg ha^−^^1^ in 2021 in RP, and 144 kg ha^−^^1^ in 2020 and 145 kg ha^−^^1^ in 2021 in CR.

In the RP system, the perennial pasture phase received fertilization during its establishment and maintenance, consistent with standard management practices of the long‐term experiment (Aguirre‐Miguez et al., 2025); however, the present study focused exclusively on the rice phase of the rotation.

All field operations used commercial‐scale machinery, and weed, pest, and disease management followed national best practices (ACA, 2018). Grain yields averaged 11.9 ± 1.5 Mg ha^−^^1^ in RP and 10.5 ± 0.6 Mg ha^−^^1^ in CR. Rice straw was retained on the soil surface after harvest, as is standard in Uruguayan rice systems.

### 2.3 Water sampling and analysis

Water samples were collected weekly over 18 weeks during each growing season (2020 and 2021), totaling 30 sampling dates per year. The schedule was designed to capture high temporal resolution during critical periods, with increased sampling frequency during early flooding and immediately after fertilization. In the first week after flooding, five sampling events were conducted; from weeks 2 to 12, sampling occurred twice weekly, except after urea application at panicle initiation, when it increased to four times per week. From week 13 to 18, samples were collected weekly. Irrigation water (IW) samples were taken weekly from the main canal supplying all plots; a total of 13 IW samples were collected per season.

Composite samples were collected at mid‐depth of the PPW (≈10 cm), using prewashed 500 mL polyethylene bottles between 9:00 a.m. and 11:00 a.m., transported on ice, and processed within 2 h. In situ water temperature was measured with an Oakton pH 150 meter.

In the laboratory, pH and electrical conductivity (EC, mS cm^−^^1^) were determined with a HANNA EDGE multiparameter meter. Total suspended solids (TSS, mg L^−^^1^) were measured following APHA (1985). Unfiltered aliquots were used for total N (TN) and TP, while the remaining sample was vacuum‐filtered (0.45 µm) to obtain dissolved fractions: dissolved total P (DTP), dissolved reactive P (DRP = PO_4_^3^^−^–P), dissolved total N (DTN), nitrate (NO_3_^−^–N), and ammonium (NH_4_^+^–N).

Both filtered and unfiltered subsamples were frozen (−20°C) until analysis, following recommended holding times. Digestions for TN, TP, DTP, and DTN followed Valderrama (1981). P fractions were quantified by Murphy and Riley (1962), N fractions by Müller and Widemann (1955), and NH_4_^+^–N by Koroleff (1970). The detection limit for NH_4_^+^–N was 10 µg L^−^^1^. All nutrient analyses were performed spectrophotometrically using a UV–Vis Genesys 150 spectrophotometer (Thermo Fisher Scientific) with a wavelength accuracy of ±0.5 nm and repeatability <±0.2 nm. All procedures followed standard QA/QC protocols to ensure analytical accuracy and reproducibility.

Derived nutrient fractions were calculated as follows: dissolved organic P (DOP) = DTP − DRP, particulate P (PP) = TP − DTP, dissolved organic N (DON) = DTN − (NH_4_^+^ + NO_3_^−^), and particulate N (PN) = TN − DTN.

### 2.4 Statistical analysis

Treatment, year, and time effects on water quality parameters were analyzed using linear mixed‐effects (LME) models in SAS Studio (SAS Institute Inc.). Fixed effects included year, treatment, week after flooding, and their interactions, while block (nested within treatment and year) was random. Repeated measures were modeled with an autoregressive correlation structure [AR(1)]. Adjusted means and interaction slices were estimated using the post‐linear model (PLM) procedure with Tukey's test (*α* = 0.05).

For nutrient fractions, a separate LME model replaced the week with the flooding phase as a categorical factor derived from temporal trends. Model residuals were checked for normality and homoscedasticity.

Principal component analysis (PCA) was performed in R (version 4.4.1; Posit Software) using the prcomp function on centered and scaled data to explore treatment‐phase clustering. Confidence ellipses were plotted using factoextra, and model‐adjusted means with standard errors (SEs) were visualized in ggplot2.

IW data were included in figures and PCA for comparison, but excluded from inferential statistics due to lack of replication. Results are presented as mean ± SE unless stated otherwise.

## 3 RESULTS

### 3.1 Nutrient concentrations and physicochemical parameters in PPW

Water quality parameters in PPW showed marked temporal variation throughout the rice flooding period. Most were significantly influenced by the week of evaluation and its interactions, while treatment and year effects were less consistent (Table 2).

**TABLE 2:** Adjusted means (± standard error [SE]) and analysis of variance (ANOVA) *p*‐values for nutrient concentrations and physicochemical parameters in paddy ponding water by year and treatment.

Effect	TN (mg L^−1^)	TP (mg L^−1^)	pH	EC (µS cm^−1^)	Temp. (°C)	TSS (mg L^−1^)
						
**2020**						
CR	5.07 ± 0.49	1.35 ± 0.48a	7.06 ± 0.03a	172 ± 7.2a	23 ± 0.29	30 ± 5.3
RP	4.00 ± 0.49	0.63 ± 0.49c	6.96 ± 0.03a	125 ± 7.2b	23 ± 0.29	29 ± 5.3
**2021**						
CR	4.38 ± 0.53	1.03 ± 0.47b	6.64 ± 0.03b	124 ± 7.2b	22 ± 0.29	32 ± 5.5
RP	4.16 ± 0.55	0.70 ± 0.47c	6.7 ± 0.03b	126 ± 7.2b	23 ± 0.28	41 ± 5.4
**ANOVA** ^a^						
Year	ns	ns	<0.01	0.02	ns	ns
Block	ns	ns	ns	ns	ns	ns
Trat	ns	<0.01	ns	0.02	ns	ns
Year × trat	ns	0.02	ns	0.02	ns	ns
Week	<0.01	<0.01	<0.01	<0.01	<0.01	ns
Trat × week	ns	<0.01	ns	<0.01	ns	ns
Year × week	<0.01	ns	<0.01	<0.01	<0.01	ns
Trat × year × week	ns	ns	ns	<0.01	ns	ns

Abbreviations: CR, continuous rice; EC, electrical conductivity; ns, not significant; RP, rice–pasture; Temp., temperature; TN, total nitrogen; TP, total phosphorus; Trat, treatment; TSS, total suspended solids.

^a^ Only significant effects (*p* ≤ 0.05) are shown in the ANOVA results.

Among all variables, TP exhibited the most pronounced treatment response, with significantly higher concentrations under CR than under RP. In contrast, TN and temperature were not affected by treatment or year, but both showed year‐by‐week interactions, indicating different temporal patterns between years. pH differed slightly between years, and EC responded to a combination of factors, showing significant interactions involving treatment, year, and week. TSS remained stable across treatments and years.

These results emphasize the importance of temporal dynamics in flooded rice systems and suggest that nutrients and physicochemical parameters cannot be fully interpreted without considering how they evolve throughout the flooding cycle.

#### 3.1.1 Nutrient dynamics in PPW and IW

**FIGURE 2:**
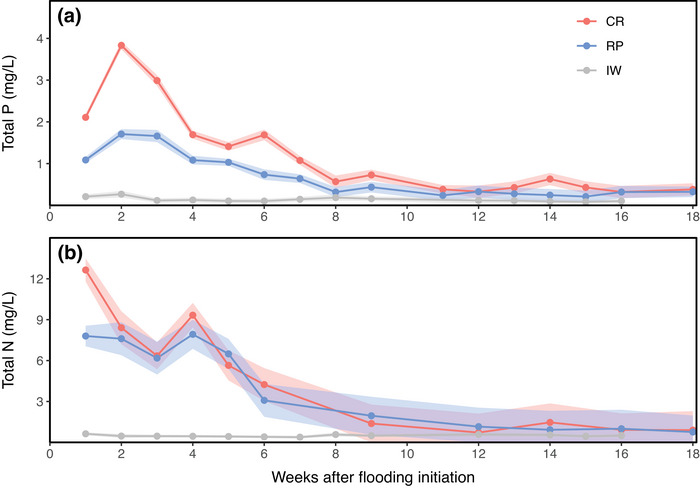
Variations in nutrient concentrations in paddy ponding water during 18 weeks of flooded rice, and in irrigation water (IW) until the end of irrigation (week 16). (a and b) Total phosphorus (TP) and total nitrogen (TN) for continuous rice (CR) and rice–pasture (RP) rotation treatments, respectively. The data represent average concentrations for the 2021 and 2022 rice growing seasons. The thicker line represents the mean for the treatments, while the shaded area around each line indicates the standard error.

Figure 2a shows the temporal evolution of mean TP concentrations in CR and RP treatments during the 18‐week flooding period of the rice crop. Significant differences between treatments were observed during the first 7 weeks (*p* < 0.01), with higher TP concentrations in CR.

During the first 2 weeks, both treatments exhibited an increase in TP concentrations, reaching peaks approximately 12 days after flooding began, with maxima of 3.84 ± 0.10 and 1.69 ± 0.12 mg L^−^^1^ for CR and RP, respectively. From the peak at week 2 to week 8, TP concentrations decreased by approximately 86% in CR and 81% in RP. From week 8 onward, TP levels in PPW stabilized at 0.59 ± 0.14 mg L^−^^1^ for CR and 0.32 ± 0.14 mg L^−^^1^ for RP, with no significant differences between treatments during this period.

Regarding TN, a trend similar to that of TP was observed (Figure 2b). However, significant differences between treatments occurred only during the first week after flooding, when TN peaked at 12.64 ± 0.84 mg L^−^^1^ in CR and 7.93 ± 1.06 mg L^−^^1^ in RP (*p* < 0.01). This initial peak likely resulted from the urea topdressing applied 1 day before flooding. A second TN peak occurred in week 4, following the second urea application at the primordium stage. From week 1 to 3, concentrations decreased by 39%, while from week 4 to 6, the reduction reached 57%. TN concentrations began to stabilize after week 6, averaging 1.10 ± 0.96 mg L^−^^1^ through the end of the monitoring period. Notably, this stabilization occurred about 2 weeks earlier than that observed for P.

IW from the Olimar River consistently exhibited lower TP and TN concentrations compared to those recorded in the evaluated treatments. Concentrations in IW remained around 0.14 ± 0.06 mg TP L^−^^1^ and 0.49 ± 0.12 mg TN L^−^^1^.

#### 3.1.2 pH and electrical conductivity in paddy ponding and irrigation water

**FIGURE 3:**
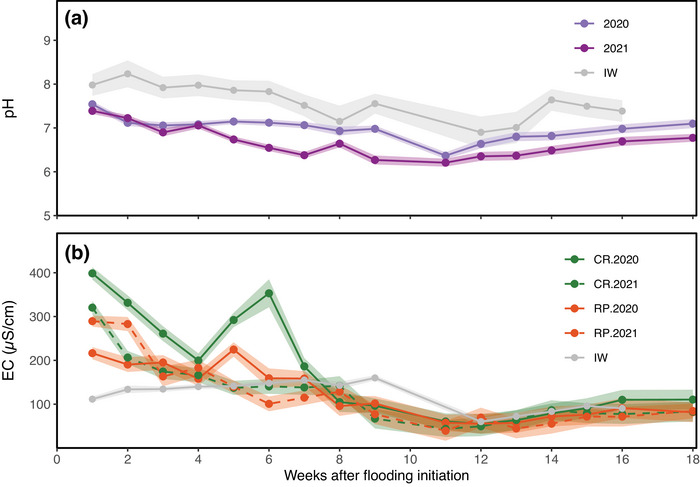
Variations in (a) pH levels and (b) electrical conductivity (EC) in the paddy ponding water during the 18 weeks of flooded rice, and in irrigation water (IW) until the end of irrigation (week 16). For pH, comparisons are made between 2020 and 2021 rice growing seasons, while for EC, the behavior of continuous rice (CR) and rice–pasture (RP) rotation treatments is compared within each year. The thicker line represents the mean for the treatments, while the shaded area around each line indicates the standard error.

Figure 3a shows the temporal dynamics of pH in PPW. During the first 4 weeks, pH remained similar between years, with a mean of 7.19 ± 0.04. From week 5 onward, significant differences appeared and persisted through the end of the monitoring period, except in week 11, when both years reached their lowest values (6.29 ± 0.06). The average pH of IW was 7.60 ± 0.11, indicating that conditions within the rice paddies acidified the water during flooding.

The analysis of EC in PPW revealed significantly higher values in CR during the first 6 weeks of 2020 (305 ± 32 µS cm^−^^1^) compared to all other treatment–year combinations (173 ± 31 µS cm^−^^1^) (Figure 3b). From week 8 onward, differences were no longer evident, with EC stabilized at an average of 135 ± 35 µS cm^−^^1^. IW exhibited an average EC of 119 ± 6.8 µS cm^−^^1^ throughout the study period, showing noticeably lower values than all treatments during the first 3 weeks.

Based on the LME analysis, a shift in the behavior of water quality parameters was identified between weeks 6 and 8 during the 18‐week rice flooding period, suggesting week 7 as a potential breakpoint.

### 3.2 Water quality differentiation across temporal phases

**FIGURE 4:**
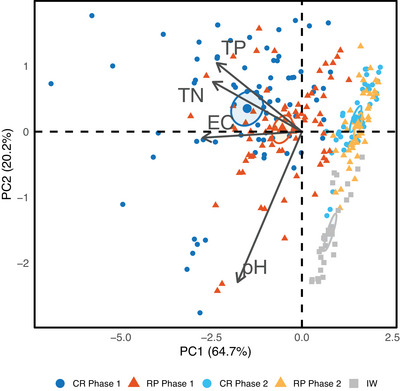
Principal component analysis (PCA) illustrating the relationships between water quality parameters (total nitrogen [TN], total phosphorus [TP], pH, and electrical conductivity [EC]) in paddy ponding water under continuous rice (CR) and rice–pasture (RP) rotation treatments, and in the irrigation water (IW). Phase 1 represents the early flooding period (weeks 1–7), while Phase 2 represents the late flooding period (weeks 8–18). Arrows indicate the direction and magnitude of variable influence on sample distribution. Ellipses represent 95% confidence intervals around the group centroids (mean positions). Percentages on axes indicate the proportion of variance explained by each principal component.

Figure 4 shows the PCA results, highlighting the clustering of water samples from rice field treatments and IW across two distinct flooding phases: Phase 1 (weeks 1–7) and Phase 2 (weeks 8–18).

The PCA explained 85% of the total variability in water quality, with 65% attributed to the first principal component (PC1). TP, TN, and EC were the main contributors to PC1 differentiation, while pH played a secondary role associated with PC2.

Phase 1 samples from both treatments clustered on the left side of PC1, whereas those from Phase 2 were positioned on the right, showing that time was the primary driver of water quality variability and highlighting Phase 1 as the period of greatest nutrient and ionic enrichment. Within Phase 1, CR and RP were clearly separated in the multivariate space, with CR showing greater dispersion and stronger association with higher nutrient concentrations. In Phase 2, the overlap of 95% confidence ellipses around group centroids indicated weaker differentiation between treatments. The spatial distribution of samples suggested a temporal shift in physicochemical properties, with reduced contributions of TP, TN, and EC to treatment separation.

To further interpret PCA patterns, the temporal trends of individual parameters (Sections 3.1.1 and 3.1.2) were considered. Mean TP concentrations in PPW were approximately threefold lower in Phase 2 than in Phase 1, TN about sevenfold lower, and EC nearly halved. IW consistently showed lower nutrient and EC concentrations than PPW, particularly in Phase 1, when TP in IW was 15‐fold and ninefold lower than in CR and RP, respectively, and TN about 19‐fold lower. Although these differences diminished in Phase 2, they still contributed to group separation in the PCA space: TP in treatments remained roughly fivefold higher than in IW, and TN about twice as high. Mean EC in CR during Phase 1 was twice that of IW, while in other treatment–phase combinations, EC averaged 22% higher than in IW.

### 3.3 Effects of treatment and phase on P and N fractions in paddy water

**FIGURE 5:**
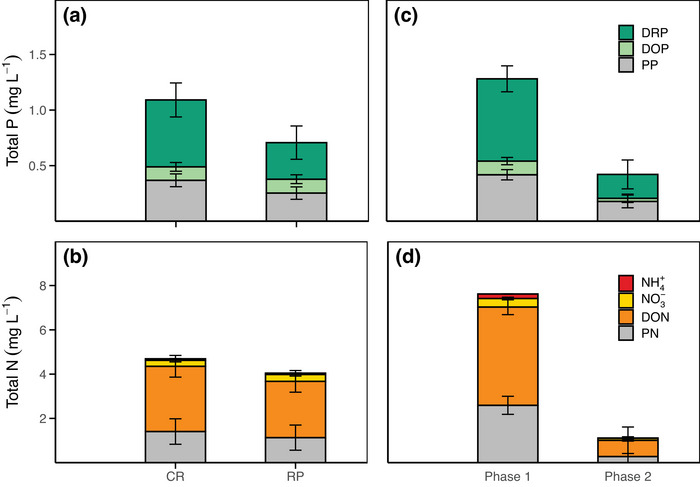
Distribution of P and N fractions in paddy ponding water. (a and b) Continuous rice (CR) and rice–pasture (RP) rotation treatments comparison. (c and d) Distribution between Phase 1 (weeks 1–7) and Phase 2 (weeks 8–18). P fractions include particulate P (PP), dissolved reactive P (DRP), and dissolved organic P (DOP). N fractions include ammonium (NH_4_^+^–N), nitrate (NO_3_^−^–N), dissolved organic N (DON), and particulate N (PN). Bar height represents total P (TP) or total N (TN) concentration, with color segments indicating the relative contribution of each fraction. Error bars represent the 90% confidence interval (CI 90%).

Figure 5 shows the distribution of P and N fractions in PPW across treatments and flooding phases. *p*‐values and significance levels from analysis of variance are provided in Tables S2 (concentrations) and S3 (fractional contributions). As no significant treatment–phase interactions were detected, treatments and phases are presented separately to highlight their individual effects.

Dissolved P and N forms predominated in PPW, with no significant differences between treatments. DTP accounted for 66% of total TP, while PP comprised the remaining 34%. DRP was the dominant fraction, but its contribution was significantly higher in CR (52%) compared to RP (45%; *p* = 0.04). Conversely, DOP was greater in RP (20%) than in CR (16%; *p* = 0.04). For N, DTN represented 71% of TN, with DON as the predominant form (63% of TN), and no significant differences were observed between treatments.

Regarding temporal dynamics, the contribution of dissolved fractions varied significantly across the flooding phases. Although both DTP and PP concentrations decreased markedly from Phase 1 to Phase 2, the proportion of DTP dropped from 70% to 63% (*p* < 0.01), leading to a relative increase in PP share from 30% to 37% (*p* < 0.01). Within the dissolved pool, the dominant form of DRP decreased from 53% in Phase 1 to 45% in Phase 2 (*p* < 0.05), while DOP showed a modest but significant increase over time (24% vs. 29%, respectively; *p* < 0.03).

For N, a significant decrease in TDN and PN concentrations was also observed. However, the proportion of TDN within TN increased from 66% in Phase 1 to 75% in Phase 2 (*p* < 0.04), and PN decreased from 34% to 25%, respectively (*p* < 0.01). Among dissolved fractions, DON remained the predominant N form across both phases, averaging 87% of TN. DON concentrations were significantly higher in Phase 1 compared to Phase 2, with average values of 3.63 ± 0.21 and 0.63 ± 0.36 mg L^−^^1^, respectively (*p* < 0.01).

For the dissolved inorganic N (DIN) fraction, NO_3_^−^–N increased from 5% in Phase 1 to 8% in Phase 2 (*p* < 0.01), whereas NH_4_^+^–N decreased significantly from 4% to 0.61% (*p* < 0.01). NO_3_^−^–N concentrations remained consistently low across treatments and phases, and did not show sharp increases at any point during the monitoring period. NH_4_^+^–N concentrations peaked during the first week after flooding and the initial topdressing, reaching 1.82 ± 0.23 mg L^−^^1^, and again following the second topdressing in week 4 (1.26 ± 0.25 mg L^−^^1^). After each urea application, NH_4_^+^–N concentrations declined rapidly, with an average reduction of 70% within 1 week.

## 4 DISCUSSION

### 4.1 Influence of land use intensification on paddy water quality

The findings of this study indicate that intensification increased TP concentrations in PPW, particularly during the first 7 weeks after flooding, while TN concentrations and pH remained unaffected. EC showed inconsistent patterns, likely reflecting interactions between management and interannual variability.

Lower pH values in PPW compared to slightly alkaline IW reflected the influence of moderately acidic paddy soils (pH ≈ 5.8) and biogeochemical shifts triggered by flooding. Respiration and organic matter decomposition, together with redox transformations, contributed to floodwater acidification (Yue & Tseng, 2019). Although land use intensity did not affect pH, climatic variability may modulate this response.

Nutrient concentrations in PPW reflect both recent fertilization and legacy nutrients (Kleinman et al., 2011). The intensified CR system demanded substantially higher P and N inputs, averaging five times more P_2_O_5_ and 69% more N than RP. Although both systems supplied similar amounts of plant‐available P, CR relied primarily on fertilizer inputs, whereas RP likely benefited from residual P accumulated during pasture periods. As a result, TP concentrations were higher in CR, accompanied by a greater relative proportion of DRP, a more bioavailable and mobile form that can enhance eutrophication risk by promoting algal blooms (Withers et al., 2017).

In contrast, TN concentrations were similar across systems, despite higher N inputs in CR, suggesting strong internal cycling. Biological N fixation by legumes in pastures and cover crops likely contributed to N supply (Hussain et al., 2025; Xing et al., 2023). Including legumes sustains yields while reducing synthetic N needs and runoff losses (Cai et al., 2018), and rice straw return further enhances soil nutrient balance (Y. Li et al., 2023; Macedo et al., 2021). The limited differences in TN between CR and RP may also reflect other biotic or abiotic mechanisms, such as gaseous N losses (Y. Feng et al., 2004) or uptake by floating vegetation (*Lemna minor*; M. Wang et al., 2023), which were not quantified in this study. Future research could investigate these processes to better understand N dynamics under intensified management.

### 4.2 Temporal dynamics of water quality in flooded rice

Water quality in PPW followed a biphasic pattern, with an initial flooding phase characterized by high and variable TP, TN, and EC, followed by stabilization after about 7 weeks. The early phase reflected rapid solute mobilization from soils, fertilizers, and residues accumulated during the dry period (Zimmerman & Kaleita, 2017). As the season progressed, EC declined and stabilized near IW levels, suggesting a shift toward ionic equilibrium.

During the first 2 weeks, TP peaked, especially in CR, due to fertilization and the onset of reducing soil conditions, releasing bound P (Yan et al., 2023). Flooding overlapped with the period of maximum P release reported by Hart et al. (2004), explaining the early peaks. TP then declined through uptake, sorption, and dilution (Hua et al., 2017), stabilizing after week 8 but remaining above IW. Similar declines have been observed elsewhere, although stabilization often occurs earlier (5–20 days) after P application (L. Liu et al., 2023; Xu et al., 2020; Yan et al., 2023).

TN concentrations peaked at the onset of inundation and declined gradually throughout the flooding period, showing a slower decrease than commonly reported (Lin et al., 2025; Xu et al., 2020; Xue et al., 2014). The slower decline, despite the high solubility of urea, likely reflects progressive N release from organic matter decomposition (Z. Zhao et al., 2015). Even during late flooding (weeks 8–18), PPW maintained higher nutrient levels than IW, indicating persistent nutrient enrichment in rice field waters.

Throughout the season, dissolved fractions dominated, particularly DRP and DON. The greater DRP proportion in CR during early flooding reflects fertilizer‐derived P, which is lost mainly in soluble forms under reducing conditions (Hart et al., 2004; Zhang et al., 2007). This is consistent with findings that DTP dominates TP losses from flooded rice rotated with upland crops (Hua et al., 2017; Yan et al., 2023). The CR system maintained higher DRP:TP ratios even as overall water quality converged between systems, suggesting that management history and soil chemistry in the intensified system enhance P mobilization. The lower extractable soil P in CR implies that this enrichment reflects greater short‐term release capacity rather than larger soil pools.

Similarly, DON dominated N forms in both systems, representing a larger TN share than inorganic forms. This contrasts with studies reporting DIN dominance (69%–91%) in runoff, while DON and PN are minor fractions (X. Wang et al., 2007). The prevalence of DON underscores the role of organic matter cycling in flooded systems, where residue and soil organic matter decomposition release substantial organic N (J. Feng & Zhu, 2021; Williams et al., 2010). Higher DON concentrations in Phase 1 likely reflect the rapid mineralization of labile organic compounds from crop residues at flooding onset. Unlike NH_4_^+^, DON is less reactive yet more persistent, contributing to sustained N availability and legacy N accumulation in aquatic environments (McCallum et al., 2021). The dominance of DON therefore suggests that microbial mineralization and residue turnover govern N dynamics more than direct fertilizer inputs, reinforcing the importance of managing organic residues and cover crops to control DON export and mitigate downstream N accumulation.

NH_4_^+^–N responded directly to urea fertilization, peaking immediately after application and declining rapidly through hydrolysis and volatilization (Dong et al., 2012). Unlike many studies reporting NH_4_^+^–N dominance in paddy floodwater under synthetic fertilizer regimes (Cui et al., 2020; Qiao et al., 2022), our results highlight a more prominent role of organic N forms, particularly during the early flooding phase. NO_3_^−^–N concentrations remained consistently low, as expected under anoxic conditions that suppress nitrification and favor gaseous N losses (Y. Feng et al., 2004).

### 4.3 Implications for nutrient management and water quality protection

P emerged as the nutrient most sensitive to land use intensification in flooded rice systems, representing a potential environmental vulnerability. The early flooding stage constitutes a critical window when episodic nutrient pulses, particularly DRP, NH_4_^+^–N, and DON, may cause short‐lived but significant downstream impacts (Dong et al., 2012; X. Zhao et al., 2009).

The persistence of a higher DRP fraction in CR, despite lower soil P, suggests that intensified rice systems and their associated nutrient management influence both nutrient stocks and their reactivity under flooding. Such functional legacy effects underscore the importance of nutrient form and mobilization potential, not only concentration, when assessing intensification risks.

These findings are particularly relevant to current intensification trends in Uruguay, where shorter pasture periods and greater crop diversification reflect a gradual departure from traditional RP systems. Although CR is not common in the country, rotations that maintain pasture phases within their current nutrient management frameworks are associated with greater nutrient retention and lower P availability under flooding. Preserving this rotational component may therefore contribute to protecting water quality under future intensification scenarios.

Both systems achieved high yields, yet pasture integration provides long‐term co‐benefits beyond productivity. Long‐term studies at this platform show that RP rotations enhance soil carbon sequestration (Macedo, Roel, Ayala, et al., 2022) and improve N and energy use efficiency, stability, and environmental performance (Macedo, Roel, Velazco, et al., 2022). Our results extend this evidence by indicating that, under the nutrient management conditions evaluated, RP rotations are associated with lower TP and DRP levels in PPW, contributing to a reduced eutrophication potential. Maintaining pasture phases within rice‐based rotations may therefore contribute to strengthening both environmental and agronomic sustainability in temperate rice systems.

P rates in the CR system were defined based on objective, long‐term nutrient management criteria aimed at sustaining system productivity rather than representing overapplication. Under intensified systems with higher fertilizer inputs, these results highlight that increased nutrient availability is an inherent consequence of land use intensification and therefore requires careful management to minimize nutrient losses.

Balancing productivity with water quality protection requires precise nutrient timing. Adjusting P application, either earlier, in split doses, or by incorporating it into the soil rather than broadcasting, may reduce flooding losses (González Jiménez et al., 2019; Zeng et al., 2021). These approaches should be evaluated under local conditions to ensure yield stability and avoid shifting nutrient losses to other stages of the cropping cycle or rotation. Complementary measures such as controlled irrigation, drainage recycling, or vegetated ditches can further mitigate nutrient losses when feasible within current infrastructure (J. Liu et al., 2025; Xiong et al., 2015).

From a management perspective, priority actions include the following: (1) aligning P fertilization to avoid peak loss periods in weeks 1–7 of flooding, (2) maintaining or reintroducing perennial pastures to enhance nutrient retention, and (3) fine‐tuning irrigation scheduling to prevent overflow events.

Incorporating these practices into extension programs and on‐farm decision tools could support the SI of rice‐based systems while minimizing eutrophication risks in connected aquatic ecosystems.

### 4.4 Study limitations and scope

This study examines water quality responses under contrasting land use intensities in rice‐based systems managed under realistic field conditions, drawing on data from an established experimental platform. The experiment includes plots large enough to be operated with commercial‐scale machinery, aiming to replicate real production processes. Nevertheless, several limitations should be considered when interpreting the results.

First, the comparison of land use intensities did not encompass the full range of rice‐based rotation alternatives feasible in production systems, including different crop or pasture sequences. Instead, the analysis focused on a strongly contrasting scenario represented by CR, which, although uncommon in Uruguay, provides a useful reference for assessing water quality responses under intensified land use conditions.

Second, fertilization strategies differed between the compared rotations, reflecting their contrasting nutrient requirements and resulting in markedly different P and N application rates. Consequently, the observed water quality responses represent the combined effect of system characteristics and nutrient management, and the relative contribution of crop frequency and fertilization cannot be fully disentangled within the constraints of the long‐term experimental design. In this context, the water quality benefits observed in the RP should not be interpreted as an inherent effect of pasture inclusion per se, but rather as the outcome of pasture phases operating within the specific nutrient management framework applied. Therefore, the results presented here are conditioned by the management strategies under which these systems were implemented and are most applicable to rice fields managed under comparable rotational structures and fertilization regimes.

Although this study provides detailed insights into nutrient dynamics in PPW under contrasting land use intensities, direct measurements of surface runoff were not conducted. Previous estimates indicate that runoff can represent approximately 11% of total inflows (Rivero et al., 2025), highlighting its potential relevance during heavy rainfall events and the importance of precise water control during early flooding. Future research should integrate runoff measurements and additional rotation phases (pasture, fallow, cover crops, and dry‐seeded rice) to identify other critical periods for nutrient loss mitigation.

Additional limitations include the focus on continuous no‐tillage systems, which restricts extrapolation to conventionally tilled rice systems. Expanding monitoring strategies to include a larger number of field replicates and a broader range of conditions would strengthen the robustness and generalization of these findings.

Despite these limitations, a major strength of this study lies in its high temporal resolution, which enabled the identification of consistent and robust patterns in nutrient dynamics in PPW across contrasting systems. Independent of land use intensity, both systems exhibited pronounced increases in N and P concentrations during the early flooding period, revealing critical windows of vulnerability for nutrient mobilization. Moreover, the contrasting temporal behavior of P and N between systems provides novel insight into how intensified rice production alters nutrient dynamics and chemical forms under flooded conditions—insights that cannot be captured through static measurements alone. These findings underscore the importance of temporal monitoring for informing nutrient and water management strategies in irrigated rice systems.

## 5 CONCLUSIONS

This study evaluated PPW quality responses under contrasting land use intensities in temperate rice‐based systems managed under realistic field conditions. Differences in nutrient concentrations and speciation in floodwater reflect the integrated response of production systems as implemented in long‐term experimental platforms, in which crop frequency, soil legacy effects, and fertilization practices operate jointly.

Under intensified rice production, higher P concentrations and a greater proportion of DRP were observed in PPW. These patterns emerged under system‐specific fertilization schemes representative of each production system, indicating that increased nutrient mobilization can occur as a consequence of intensified land use and associated management strategies, rather than solely from non‐recommended fertilizer applications. Accordingly, water quality responses should be interpreted in the context of the combined effects of cropping intensity and fertilization approach.

Across both systems, pronounced increases in N and P concentrations during the early flooding period identify a critical window of vulnerability for nutrient mobilization, regardless of land use intensity. Differences in nutrient speciation further indicate that intensification alters not only nutrient concentrations but also their biogeochemical behavior under flooded conditions.

Overall, the results highlight that contrasting production systems with distinct fertilization strategies can differ substantially in their water quality outcomes, underscoring the need for adaptive nutrient and water management, particularly during early flooding, to sustain rice productivity while protecting freshwater resources.

## AUTHOR CONTRIBUTIONS

**G. Cantou**: Conceptualization; data curation; formal analysis; investigation; methodology; writing—original draft; writing—review and editing. **P. González‐Barrios**: Data curation; formal analysis; writing—review and editing. **I. Furtado**: Investigation. **A. Roel**: Conceptualization; investigation; writing—review and editing.

## CONFLICT OF INTEREST STATEMENT

The authors declare no conflicts of interest.

## Supporting information




**Table S1**. Fertilizer applications by treatment and year.
**Table S2**. Adjusted means (±SE) and ANOVA p‐values for concentrations of phosphorus and nitrogen fractions in paddy ponding water by treatment and flooding phase.
**Table S3**. Adjusted means (±SE) and ANOVA p‐values for the proportional contribution of phosphorus and nitrogen fractions in paddy ponding water by treatment and flooding phase.
