# Supplementary material for: Paddy ponding water quality responses to land use intensity in flooded rice systems in Uruguay
Source: J Environ Qual. 2026 Feb 27;55(2):e70156. doi: 10.1002/jeq2.70156 (PMC12947055; doi:10.1002/jeq2.70156)
Supplement: Supplementary file 1 — Table S1. Fertilizer applications by treatment and year. Table S2. Adjusted means (±SE) and ANOVA p‐values for concentrations of phosphorus and nitrogen fractions in paddy ponding water by treatment and flooding phase. Table S3. Adjusted means (±SE) and ANOVA p‐values for the proportional contribution of phosphorus and nitrogen fractions in paddy ponding water by treatment and flooding phase. [file JEQ2-55-0-s001.docx]

Supplementary material

Table S1. Fertilizer applications by treatment and year.

| **Year** | **Treatment** | **Basal fertilizer** | | **N at tillering** | **N at panicle** | **Total P₂O₅** | **Total N** |
| --- | --- | --- | --- | --- | --- | --- | --- |
|  |  | **In-line** | **Broadcast** | ----- **Broadcast (kg ha⁻¹)** ----- | | ----------------- **(kg ha⁻¹)** ----------------- | |
| 2020 | RP | 60 kg ha⁻¹ of 5-25-25-2 | 108 kg ha⁻¹ of 0-0-60 | 38 | 35 | 15 | 76 |
| 2020 | CR | 60 kg ha⁻¹ of 5-25-25-2 | 176 kg ha⁻¹ of 0-40-0-4 and 49 kg ha⁻¹ of 0-0-60 | 95 | 46 | 85 | 144 |
| 2021 | RP | 78 kg ha⁻¹ of 9-25-25-3 | — | 46 | 55 | 20 | 108 |
| 2021 | CR | 78 kg ha⁻¹ of 9-25-25-3 | 182 kg ha⁻¹ of 0-40-0-4 and 78 kg ha⁻¹ of 0-0-60 | 92 | 46 | 92 | 145 |

Notes: Basal P and K fertilizers were applied at planting using in-line and broadcast application. Nitrogen was broadcast as urea in two splits: at tillering on dry soil immediately before flooding, and at panicle initiation on flooded soil. Fertilizer formulations correspond to N-P₂O₅-K₂O-S ratios. Both treatments followed best management practices under commercial-scale conditions.

Table S2. Adjusted means (±SE) and ANOVA p-values for concentrations of phosphorus and nitrogen fractions in paddy ponding water by treatment and flooding phase.

| **Effect** | **PHOSPHORUS FRACTIONS** | | | |  | **NITROGEN FRACTIONS** | | | | |  |
| --- | --- | --- | --- | --- | --- | --- | --- | --- | --- | --- | --- |
|  | **DTP** | **PP** | **DRP** | **DOP** |  | **DTN** | **PN** | **NO₃⁻-N** | **NH₄⁺-N** | **DON** | |
| Treatment |  |  |  |  |  |  |  |  |  |  | |
| CR | 0.94 ± 0.13 | 0.37 ± 0.04 | 0.76 ± 0.09 | 0.15 ± 0.02 |  | 3.05 ± 0.32 | 1.73 ± 0.35 | 0.20 ± 0.04 | 0.02 ± 0.01 | 2.37 ± 0.30 | |
| RP | 0.57 ± 0.12 | 0.26 ± 0.03 | 0.40 ± 0.09 | 0.15 ± 0.02 |  | 2.29 ± 0.33 | 1.53 ± 0.34 | 0.23 ± 0.04 | 0.02 ± 0.01 | 1.89 ± 0.30 | |
| Phase |  |  |  |  |  |  |  |  |  |  | |
| 1 | 1.03 ± 0.10 | 0.47 ± 0.03 | 0.76 ± 0.07 | 0.20 ± 0.02 |  | 4.63 ± 0.24 | 2.99 ± 0.25 | 0.28 ± 0.04 | 0.04 ± 0.01 | 3.63 ± 0.21 | |
| 2 | 0.48 ± 0.11 | 0.16 ± 0.03 | 0.41 ± 0.08 | 0.10 ± 0.02 |  | 0.71 ± 0.39 | 0.27 ± 0.42 | 0.14 ± 0.04 | 0.00 ± 0.01 | 0.63 ± 0.36 | |
| ANOVA^†^ |  |  |  |  |  |  |  |  |  |  | |
| Year | ns | ns | ns | ns |  | ns | ns | ns | ns | ns | |
| Block | ns | ns | ns | ns |  | ns | ns | ns | ns | ns | |
| Trat | ns | ns | ns^‡^ | ns |  | ns | ns | ns | ns | ns | |
| Trat*Year | ns | ns | ns | ns |  | ns | ns | ns | ns | ns | |
| Phase | <0.01 | <0.01 | <0.01 | <0.01 |  | <0.01 | <0.01 | <0.01 | <0.01 | <0.01 | |
| Trat*Phase | ns | ns | ns | ns |  | ns | ns | ns | ns | ns | |
| Year*Phase | ns | ns | ns | ns |  | ns | ns | ns | ns | ns | |
| Trat*Year*Phase | ns | ns | ns | ns |  | ns | ns | ns | ns | ns | |

^†^ Only significant effects (*P* ≤ 0.05) are shown in the ANOVA results; ns: not significant.

^‡^ The p-value was 0.07, indicating a trend toward significance.

Abbreviations: P fractions [dissolved total P (DTP), particulate P (PP), dissolved reactive P (DRP), and dissolved organic P (DOP)] and N fractions [dissolved total N (DTN), particulate N (PN), nitrate-N (NO₃⁻–N), ammonium-N (NH₄⁺–N), and dissolved organic N (DON)].

Definitions: Flooding phase (Phase 1: weeks 1–7; Phase 2: weeks 8–18) and treatment [Continuous Rice (CR) and Rice–Pasture rotation (RP)].

Table S3. Adjusted means (±SE) and ANOVA p-values for the proportional contribution of phosphorus and nitrogen fractions in paddy ponding water by treatment and flooding phase.

| **Effect** | **PHOSPHORUS FRACTIONS**^†^ | | | |  | **NITROGEN FRACTIONS**^‡^ | | | | |  |
| --- | --- | --- | --- | --- | --- | --- | --- | --- | --- | --- | --- |
|  | **%DTP** | **%PP** | **%DRP** | **%DOP** |  | **%DTN** | **%PN** | **%NO₃⁻-N** | **%NH₄⁺-N** | **%DON** | |
| Treatment |  |  |  |  |  |  |  |  |  |  | |
| CR | 68 ± 2.9 | 32 ± 2.9 | 77 ± 1.4 | 23 ± 1.4 |  | 70 ± 4.7 | 30 ± 4.7 | 8.6 ± 0.8 | 2.8 ± 0.6 | 88 ± 1.2 | |
| RP | 65 ± 2.9 | 35 ± 2.9 | 70 ± 1.4 | 30 ± 1.4 |  | 72 ± 4.6 | 28 ± 4.6 | 11.0 ± 0.8 | 2.5 ± 0.6 | 86 ± 1.2 | |
| Phase |  |  |  |  |  |  |  |  |  |  | |
| 1 | 70 ± 2.3 | 30 ± 2.2 | 76 ± 1.2 | 24 ± 1.1 |  | 66 ± 3.3 | 34 ± 3.3 | 8.4 ± 0.6 | 4.7 ± 0.5 | 86 ± 0.9 | |
| 2 | 63 ± 2.6 | 37 ± 2.6 | 71 ± 1.6 | 29 ± 1.6 |  | 75 ± 4.7 | 25 ± 4.7 | 11.2 ± 0.9 | 0.6 ± 0.8 | 88 ± 1.5 | |
| ANOVA^§^ |  |  |  |  |  |  |  |  |  |  | |
| Year | ns | ns | ns | ns |  | ns | ns | ns | ns | ns | |
| Block | ns | ns | ns | ns |  | ns | ns | ns | ns | ns | |
| Trat | ns | ns | 0.04 | 0.03 |  | ns | ns | ns | ns | ns | |
| Trat*Year | ns | ns | ns | ns |  | ns | ns | ns | ns | ns | |
| Phase | <0.01 | <0.01 | 0.05 | 0.03 |  | 0.04 | 0.04 | <0.01 | <0.01 | ns | |
| Trat*Phase | ns | ns | ns | ns |  | ns | ns | ns | ns | ns | |
| Year*Phase | ns | ns | ns | ns |  | ns | ns | ns | ns | ns | |
| Trat*Year*Phase | ns | ns | ns | ns |  | ns | ns | ns | ns | ns | |

^†^ P fractions DRP and DOP were calculated as a proportion of DTP. DTP and PP fractions were calculated as a proportion of TP.

^‡^ N fractions NO₃⁻–N, NH₄⁺–N and DON were calculated as a proportion of DTN. DTN and PN fractions were calculated as a proportion of TN.

^§^ Only significant effects (*P* ≤ 0.05) are shown in the ANOVA results; ns: not significant.

Abbreviations: P fractions [dissolved total P (DTP), particulate P (PP), dissolved reactive P (DRP), and dissolved organic P (DOP)] and N fractions [dissolved total N (DTN), particulate N (PN), nitrate-N (NO₃⁻–N), ammonium-N (NH₄⁺–N), and dissolved organic N (DON)].

Definitions: Flooding phase (Phase 1: weeks 1–7; Phase 2: weeks 8–18) and treatment [Continuous Rice (CR) and Rice–Pasture rotation (RP)].
